# Structural and functional features of asthma participants with fixed airway obstruction using CT imaging and 1D computational fluid dynamics: A feasibility study

**DOI:** 10.14814/phy2.15909

**Published:** 2024-01-07

**Authors:** Quoc Hung Nguyen, So Ri Kim, Kum Ju Chae, Gong Yong Jin, Sanghun Choi

**Affiliations:** ^1^ School of Mechanical Engineering Kyungpook National University Daegu South Korea; ^2^ Division of Respiratory Medicine and Allergy, Department of Internal Medicine Research Institute of Clinical Medicine of Jeonbuk National University–Biomedical Research Institute of Jeonbuk National University Hospital Jeonju South Korea; ^3^ Department of Radiology Research Institute of Clinical Medicine of Jeonbuk National University–Biomedical Research Institute of Jeonbuk National University Hospital Jeonju South Korea

**Keywords:** airflow distribution, and pressure drop, computational fluid dynamics, computed tomography, wall thickening

## Abstract

Asthma with fixed airway obstruction (FAO) is associated with significant morbidity and rapid decline in lung function, making its treatment challenging. Quantitative computed tomography (QCT) along with data postprocessing is a useful tool to obtain detailed information on airway structure, parenchymal function, and computational flow features. In this study, we aim to identify the structural and functional differences between asthma with and without FAO. The FAO group was defined by a ratio of forced expiratory volume in 1 s (FEV_1_) to forced vital capacity (FVC), FEV_1_/FVC <0.7. Accordingly, we obtained two sets of QCT images at inspiration and expiration of asthma subjects without (*N* = 24) and with FAO (*N* = 12). Structural and functional QCT‐derived airway variables were extracted, including normalized hydraulic diameter, normalized airway wall thickness, functional small airway disease, and emphysema percentage. A one‐dimensional (1D) computational fluid dynamics (CFD) model considering airway deformation was used to compare the pressure distribution between the two groups. The computational pressures showed strong correlations with the pulmonary function test (PFT)‐based metrics. In conclusion, asthma participants with FAO had worse lung functions and higher‐pressure drops than those without FAO.

## Graphical abstract

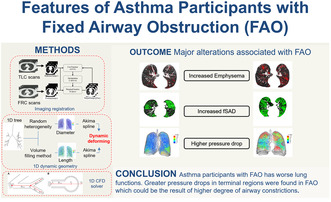

Visual summary of the current study

## 1 INTRODUCTION

Asthma is a multifactorial, heterogeneous, and chronic inflammatory disease that affects both large and small airways. Participants with asthma can present with breathlessness, chest tightness, coughing, and wheezing because of different types of airflow obstruction and airway hyperresponsiveness. Although there has been a decrease in hospitalization and death among participants with asthma, its high prevalence remains a significant problem for both the medical system and society at large (Global Initiative for Asthma, 2022). Severe asthma (SA) is a life‐threatening condition that affects only a small percentage of participants with asthma but accounts for >60% of total asthma‐related medical expenses, thus imposing a heavy socioeconomic burden. According to the Global Initiative for Asthma guidelines, SA is defined as uncontrolled asthma despite adherence to optimized high‐dose inhaled corticosteroid (ICS)‐long‐acting β‐2 agonists (LABA) therapy and treatment of contributory factors; it is also defined as asthma that worsens on decreasing high dose treatment (Global Initiative for Asthma, 2022). Fixed airway obstruction (FAO) is one of the underlying mechanisms causing persistent and severe clinical phenotypes in SA (Gaga, 2004); which is difficult to treat. SA with FAO is associated with an accelerated decline in lung function and excess morbidity (Rutting et al., 2022). In the previous several decades, extensive investigation of asthma pathogenesis has led to a better understanding of the disease and the development of a more comprehensive therapeutic approach (Dusser et al., 2007). However, in participants with SA, especially those with FAO, no currently available treatments can reverse the excessive decline in lung function or airway remodeling, which results in failure to control the disease (Calogero et al., 2018; Moore et al., 2010). Diagnostic, and therapeutic advances in SA treatment have been made, and the current consensus is that FAO in asthma is caused by airway remodeling associated with persistent inflammation; however, its risk factors and the underlying pathological mechanisms remain unclear.

Pulmonary function test (PFT) is a highly useful and practical tool used for the assessment and monitoring of participants with asthma, especially those with SA (Miller et al., 2005; Moeller et al., 2015; Pellegrino et al., 2005). According to European Respiratory Society/American Thoracic Society guidelines (Chung et al., 2014), one of the main criteria for recognizing “uncontrolled asthma” is airflow limitation, defined as predicted FEV_1_ < 80% (prebronchodilator) in the presence of a reduced FEV_1_/FVC ratio (<0.7). Several reports have indicated that FAO is a key predictor of overall mortality in participants with asthma, however, the usage of PFTs provided limited information regarding the nature of the obstruction (Hansen et al., 1999; Lee et al., 2007; ten Brinke et al., 2001). In other works, FEV_1_ may not always be correlated with symptoms; some evidence suggests that FEV_1_ can be normal in symptomatic children with poorly controlled asthma (Hansen et al., 1999; Lee et al., 2007; ten Brinke et al., 2001). Taken together, there is a need for more effective tools to assess airflow obstruction.

Computed tomography (CT) imaging can provide structural and functional phenotypes of diseased lungs, such as those affected by asthma and chronic obstructive pulmonary disease (COPD); these images can provide more information regarding spatial features compared with those obtained using other methods such as defining four asthmatic clusters using structural and functional characteristics (Choi, Hoffman, et al., 1985), differentiating between COPD phenotypes (Galbán et al., 2012), or differently varying local volume change in asthmatic populations (Choi et al., 2013; Galbán et al., 2012). Previous studies (Choi, Yoon, et al., 1985; Yoon et al., 1985) have investigated ventilation heterogeneity, airway pressure, and flow distributions using 1D CFD simulations based on CT image data. Specifically, Choi et al. (Choi, Yoon, et al., 1985) used 4D CT imaging to demonstrate that participants with asthma have higher resistance owing to airway constriction and used a dataset based on two static CT images and a 1D airway network model to illustrate the pressure distribution during breathing. However, these studies either applied to limited numbers of participants or were not targeted to investigate the subset of participants with asthma such as ones with FAO.

QCT imaging variables and computation‐derived metrics are an integrated set of assessments for characterizing asthma. In this study, we aim to explore these imaging and post‐process metrics for asthma participants with and without FAO. We hypothesize that some imaging metrics can identify pathological phenotypes in asthma with FAO (relative to asthma without FAO) that are not visible from PFT results alone. Furthermore, we apply a dynamic 1D airway network model, which considers structural deformation during breathing, to simulate the pressure distribution within participants and compare the two groups. Finally, we check the correlation between lung function test results with CFD outputs to better understand the efficacy of the computational model in assessing asthma characteristics.

## 2 METHODS

### 2.1 Participant selection

**FIGURE 1:**
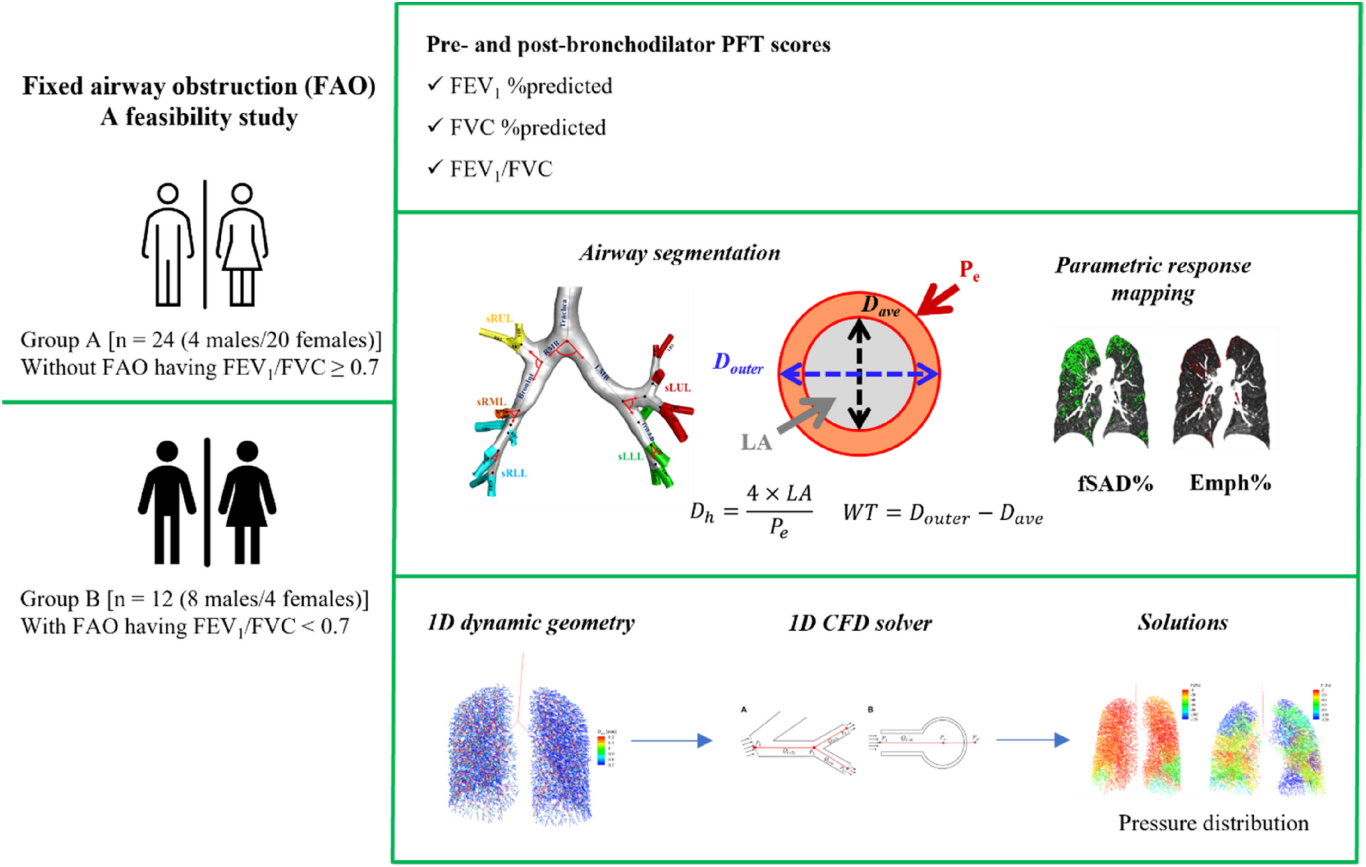
Summary diagram including studied populations; extraction of quantitative computed tomography imaging‐based features, including airway structural and lung functional features; 1D dynamic CFD model framework.

This study was retrospectively designed and approved by the Institutional Review Board of Jeonbuk National University Hospital (IRB‐2019‐07‐032‐008). The participant consent forms were obtained verbally when participant imaging and clinical data were obtained. Since this study was designed retrospectively, the written informed consent form was waived by the IRB. Overall, 163 participants with asthma undergoing inspiratory and expiratory CT from January 2015 to December 2018 were initially enrolled in the study. Enrolled participants were ≥ 18 years of age and were diagnosed with asthma by a medical doctor. All participants underwent standard treatment as recommended by international and domestic guidelines for asthma management, including ICS administration. The asthma diagnosis was confirmed by an allergy specialist (S.R.K. with 20 years of experience) based on medical history and the presence of either bronchodilator reversibility or bronchial hyperresponsiveness. The participants were excluded from the study if they met the following criteria: (1) presence of infectious lung disease, such as pneumonia or pulmonary tuberculosis, on CT imaging (*n* = 18); (2) indeterminate smoking history (*n* = 12) or currently/previously smoking (*n* = 62); (3) failure of image segmentation (*n* = 2); (4) inadequate expiration (i.e., expiratory volume/inspiratory volume > 90%) (*n* = 17); and (5) the CT scan and PFT analysis dates were obtained outside of a 6 month window (*n* = 16). A total of 36 participants with asthma remained and were included in further analysis. Based upon postbronchodilator PFT, the participants were divided into Groups A (*n* = 24) and B (*n* = 12) which included asthma participants without FAO and with FAO, respectively. This classification was based on their PFT results such that the FEV_1_/FVC ratio is less than 0.7. The selected participants are summarized in Figure 1. Results of PFT performed within 3–6 months of the CT scan were analyzed: 26 participants on CT scan date, three participants within 1 week, one participant within 1 month, three participants within 2 months, and three participants within 6 months.

### 2.2 Data extraction and acquisition

Clinical characteristics, smoking history and intensity, PFT results, and CT features were reviewed by two radiologists specializing in thoracic imaging (i.e., K.J.C. and G.Y.J., with 9 and 20 years of experience, respectively) along with the allergy specialist mentioned above. Using a 128 multidetector CT scanner (Somatom Definition Flash, Siemens Healthcare, Forchheim, Germany), inspiratory and expiratory CTs were taken under full inspiration and normal expiration. CT images were taken without iterative reconstruction. CT imaging parameters were set as follows: 110 (inspiration) and 50 (expiration) effective mAs tube current, 128 × 0.6 mm acquisition, 120 kVp tube voltage, 1.0 mm reconstruction thickness, 1.0 mm reconstruction increment, B35f reconstruction algorithm, 0.5 s rotation time, and 1.0 pitch. PFT were performed as per the guidelines of the American Thoracic Society (Graham et al., 2019). A portable spirometer (Chest Graph HI‐701, Chest Co. Ltd, Tokyo, Japan) was used to record the following values before and after participants took a bronchodilator: forced expiratory volume in 1 s (FEV_1_), FVC, and FEV_1_/FVC ratio. We used the values derived from Eom et al. (Eom & Kim, 2013) for Korean populations to compute predicted values.

### 2.3 Quantitative imaging analysis

The airways, lungs, and lobes were segmented from CT images using the airway segmentation software VIDA Apollo, version 2.0 (Vida Diagnostics, Coralville, IA, US). The mass‐preserving image registration method was used to match inspiratory and expiratory CT images (Youbing et al., 2009; Yin et al., 2009). We identified 36 segmental regions of interest (ROIs) through anatomical labeling and divided them into 19 relatively small segments (RB1–6, RB8–10, and LB1–10) and thereafter in the following five subgroups: right upper lobe (sRUL), right middle lobe (sRML), right lower lobe (sRLL), left upper lobe (sLUL), and left lower lobe (sLLL). We also selected six central airway branches, that is, the trachea, right main bronchus (RMB), bronchus intermedius (Bronint), trifurcation of the right lower lobe (TriRLL), left main bronchus (LMB), and trifurcation of the left lower bronchus (TriLLB), resulting in a total of 11 regions to investigate. Additional details regarding the segmental airways and ROIs have been previously reported (Choi, Hoffman, et al., 1985). We then extracted the structural and functional QCT‐derived variables for all airways to compare the multiscale lung structure and function parameters for each group using Mann–Whitney *U* tests. Specifically, hydraulic diameter (*D*_*h*_) and airway wall thickness (*WT*) were normalized by the predicted values of trachea diameter and wall thickness from Korean populations (Chae et al., 2021), respectively, for local structural alteration analysis, and we calculated functional variables such as functional small airway disease percentage (fSAD%) and emphysema percentage (Emph%) based on adjusted thresholds of voxel density to assess participant lung function (Choi, Hoffman, et al., 1985).

### 2.4 1D CFD model

**FIGURE 2:**
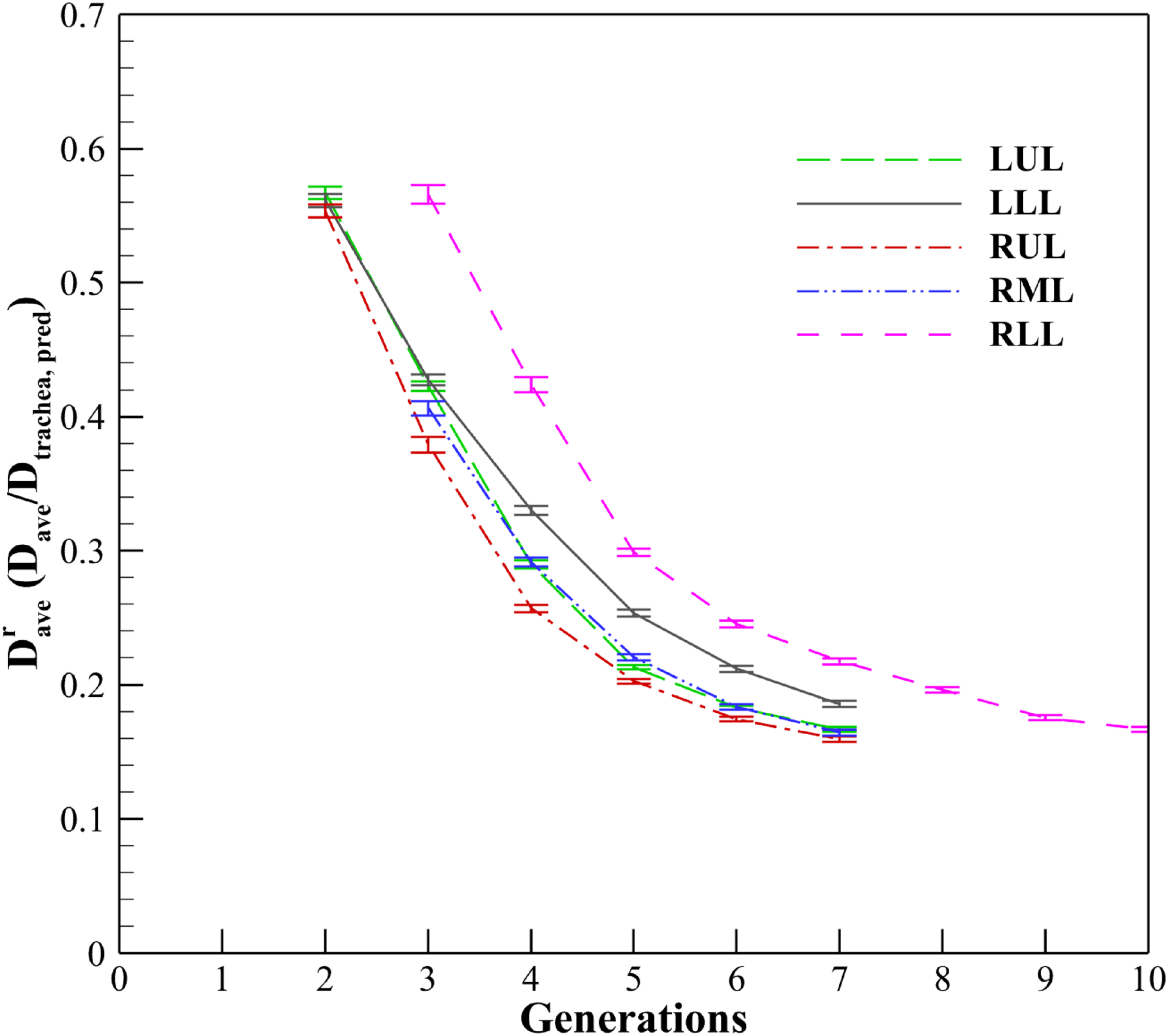
A generational mean (SE) ratio $D_{\mathrm{ave}}^{r}$ of CT‐resolved airway diameter ($D_{\mathrm{ave}}$) to predicted tracheal diameter ($D_{\text{trachea},\text{pred}}$) in 5 lobes from 222 healthy Korean participants (Chae et al., 2021)].

Since CT resolution limits reliable branch data with a diameter less than ~2 mm, it is necessary to construct physiologically reasonable branching patterns in CT‐unresolved airway regions to permit complete lung CFD simulation. To produce such branching patterns, including branch angles and lengths, we used a volume‐filling method that is found in two papers by Tawhai et al. (Howatson Tawhai et al., 2000; Tawhai et al., 1985). Regarding the airway diameter in CT‐unresolved airways, a stochastic random regional heterogeneity along with Horsfield ordering is used in each lobe. The procedure of obtaining the physiologically reasonable diameters is summarized as follows. We employed an equation introduced by Chae et al. (Chae et al., 2021) that predicts the tracheal diameters based on a population of 222 healthy Koreans as follows:

$$\begin{array}{c} D_{\text{trachea},\text{pred}}\;\left(\mathrm{mm}\right)=12.79-0.13\log\left(\mathrm{age}\right)\\ \;-5.82\log\left(\text{height}\right)*\mathrm{sex}\\ \;+3.01\log\left(\mathrm{age}\right)*\log\left(\text{height}\right),\;\left(1\right) \end{array}$$

where [sex, height, age] take the value/unit of [0 (male) or 1 (female), meter, year], and a natural log is used for the logarithmic function. This equation demonstrated significant correlation of the tracheal diameter with sex, height, and age. In this study, from the healthy Korean participants, we computed a healthy diameter ratio $D_{\mathrm{ave}}^{r}$ that is the value of $D_{\mathrm{ave}}$ divided by $D_{\text{trachea},\text{pred}}$. Figure 2 plotted the value of $D_{\mathrm{ave}}^{r}$ according to generation number and the respective five lobes including the left upper lobe (LUL), the left lower lobe (LLL), the right upper lobe (RUL), the right middle lobe (RML), and the right lower lobe (RLL). Specific values can be found in Table 1. Given the value of $D_{\mathrm{ave}}^{r}$, we could predict the average diameters $D_{\mathrm{ave},\text{pred}}$ of healthy Korean participants as per lobes and generations using the equation below:

(2) $$D_{\mathrm{ave},\text{pred}}=D_{\text{trachea},\text{pred}}\times D_{\mathrm{ave}}^{r}$$

With $D_{\mathrm{ave},\text{pred}}$, we could estimate the degree of airway constriction/dilation from CT‐resolved airways in the following equation:

(3) $$D_{\mathrm{ave}}^{*}=D_{\mathrm{ave}}/D_{\mathrm{ave},\text{pred}}$$

**TABLE 1:** Generational $D_{\mathrm{ave}}^{r}$ in five lobes from 222 healthy Korean participant*s.*

	Generation								
	2	3	4	5	6	7	8	9	10
LUL	0.567 (0.005)	0.423 (0.003)	0.290 (0.003)	0.213 (0.002)	0.183 (0.002)	0.167 (0.002)			
LLL	0.561 (0.005)	0.427 (0.004)	0.330 (0.003)	0.254 (0.002)	0.212 (0.002)	0.186 (0.002)			
RUL	0.554 (0.005)	0.379 (0.006)	0.257 (0.003)	0.203 (0.002)	0.174 (0.002)	0.159 (0.002)			
RML		0.406 (0.005)	0.292 (0.003)	0.221 (0.002)	0.183 (0.002)	0.164 (0.002)			
RLL		0.566 (0.007)	0.424 (0.006)	0.299 (0.003)	0.245 (0.002)	0.218 (0.002)	0.196 (0.002)	0.175 (0.002)	0.167 (0.002)

*Note*: Values are presented as mean (standard error, SE).

Abbreviations: LLL, left lower lobe, LUL, left upper lobe, RLL, right lower lobe, RML, right middle lobe, RUL, right upper lobe.

Note that this quantity is closer to normal ranges $D_{\mathrm{ave}}^{*}\approx 1$ in healthy participants (Choi, Yoon, et al., 1985).

We then computed the mean and standard deviation (SD) of $D_{\mathrm{ave}}^{*}$ as per lobes with the CT‐resolved airways and generated a random value from normal distribution of the mean (SD). For CT‐unresolved airways, an initial healthy diameter was first estimated using the Horsfield ordering method (Horsfield et al., 1971) and then was modified by multiplying with the random values of $D_{\mathrm{ave}}^{*}$. This stochastic approach could be considered a good approximation of CT‐unresolved airway diameters from segmental airways unless higher‐resolution CT scans are available. Meanwhile, for dynamic simulation, we interpolated node coordinates, and diameters between TLC and FRC by using the Akima spline (Yoon et al., 1985).

Choi et al. (Choi, Yoon, et al., 1985) used an isothermal energy balance equation simplified from a 3D model to a 1D model to obtain pressure and flow distributions for the whole airways. Yoon et al. (Yoon et al., 1985) later extended this model to cover the dynamic deformation of the lungs during breathing; the 1D model further integrated the effects of wall compliance and parenchymal inertance. These two previous studies demonstrated that a 1D lung CFD model could be used to assess lung function as an alternative to PFTs. In this study, the 1D model is employed to assess pressure drop, workload, pressure hysteresis with some different setups. To focus on the analysis of pressure distribution, we imposed the same values for numerical conditions, such that tidal volume (V_tidal_) and breathing period were set to 1 L and 4 s, respectively, with a sinusoidal wave form at inlets. We also imposed a fixed global compliance of $0.2\;{\left[\mathrm{cm}H_{2}O\right]}^{-1}$ (Briscoe & Dubois, 1958) instead of using a subject‐specific lung compliance model (Wongviriyawong et al., 2013).

### 2.5 Statistical analyses

Nonparametric Mann–Whitney *U* test and parametric *t*‐tests were performed to determine whether the QCT‐based and computational variables differed between Groups A and B for non‐normal and normal data, respectively. The Shapiro–Wilk tests were used to test the normality of the data. The relationships between the computational features and PFT results were then analyzed using Spearman correlation tests. All statistical analyses were performed using Python and the threshold of statistical significance was *p* < 0.05 for a total of 68 comparison tests, resulting in a false discovery rate (FDR) of 25.0%. We corrected the *p*‐values using the Benjamini‐Hochberg procedures and reported the resulting Q‐values accordingly to identify the significant features.

## 3 RESULTS

### 3.1 PFT variables

Table 2 presents the demographic and PFT information of the 36 participants with asthma included in the current study divided into participants with the reversible (Group A: *N* = 24) and fixed (Group B: *N* = 12) airway obstructions. We noted a large sex imbalance between Group A and Group B with ~16.67% males and 66.67% males, respectively. Comments on its effect on the results are discussed later. The differences in age and BMI between the two groups were negligible (*p* = 0.631, *p* = 0.518, respectively), and the %predicted values of FEV_1_ were significantly lower in Group B than Group A before (*p* = 0.006) and after (*p* = 0.013) postbronchodilator. This indicates that the participants in Group A had better lung function than those in Group B. On the other hand, the pre‐ and post‐ %predicted values of FVC were not significantly different between the two groups (*p* = 0.251, *p* = 0.694, respectively). The ratios of pre‐ and post‐ predicted FEV_1_/FVC were also significantly lower in Group B compared with Group A (*p* < 0.001) as expected due to classification criteria. The counts of airways resolved by the CT were not different between the two groups at both TLC and FRC (*p* = 0.518, *p* = 0.072, respectively).

**TABLE 2:** Demographics and pulmonary function of asthma groups: Group A (without FAO) and Group B (with FAO).

	Group A (*n* = 24)	Group B (*n* = 12)	*p*‐Value	Q‐Value
Sex, *n*, male/female	4/20	8/4		
Age, years	62.583 (11.100)	63.667 (13.089)	0.631	0.678
BMI, kg/m^2^	25.219 (4.888)	24.382 (3.819)	0.518	0.574
Prebronchodilator				
FEV_1_, % predicted	101.125 (18.755)	76.917 (20.025)	0.006	0.019
FVC, % predicted	95.792 (13.584)	87.417 (16.638)	0.251	0.329
FEV_1_/FVC, %	75.833 (5.113)	61.250 (8.476)	<0.001	<0.001
Postbronchodilator				
FEV_1_, % predicted	102.913 (17.962)	81.083 (21.284)	0.013	0.033
FVC, % predicted	96.087 (12.986)	91.500 (18.158)	0.694	0.734
FEV_1_/FVC, %	77.130 (4.404)	61.750 (7.461)	<0.001	<0.001
Total Airway Counts				
TLC	142.958 (42.398)	137.667 (54.583)	0.518	0.574
FRC	93.708 (32.678)	78.917 (38.161)	0.072	0.130

*Note*: Values are presented as mean (standard deviation, SD).

Abbreviations: BMI, body mass index, FEV_1,_ forced expiratory volume in 1 s, FVC, forced vital capacity.

### 3.2 QCT imaging variables

Table 3 shows the QCT‐derived percentages of emphysema (Emph%) and functional small airway disease (fSAD%) in total and five lung lobes of the two groups. Overall, participants in Group B had significantly increased fSAD% values in all lung lobes and increased Emph% in RLL, and total lung, confirming that Group B participants had more diseased lungs compared to those in Group A. The normalized hydraulic diameter *D*_h_* values of Group B participants were significantly larger only in trachea, RMB, BronInt, and LMB branches than those of Group A participants (Table 4), whereas there is no statistical difference of *D*_h_* in segmental airways. The normalized wall thickness WT* values of Group B participants were significantly larger in RMB, TriRLL, TriLLB, sRML, sLUL, and sLLL branches than those of Group A participants.

**TABLE 3:** QCT‐derived percentage of emphysema (Emph%) and percentage of functional small airway disease (fSAD%) in five lung lobes.

Variable	Region	Group A	Group B	*p*‐Value	Q‐value
Emph%	LUL	0.192 (0.219)	0.631 (1.103)	0.436	0.514
	LLL	0.222 (0.350)	1.951 (3.158)	0.067	0.127
	RUL	0.084 (0.101)	0.792 (2.067)	0.067	0.127
	RML	0.397 (0.445)	1.266 (2.404)	0.476	0.544
	RLL	0.176 (0.221)	1.126 (1.731)	0.004	0.015
	Total	0.195 (0.217)	1.120 (1.878)	0.010	0.028
fSAD%	LUL	5.613 (6.676)	17.236 (11.241)	<0.001	0.003
	LLL	3.228 (6.350)	16.373 (18.437)	0.001	0.005
	RUL	4.708 (6.135)	14.129 (12.925)	0.002	0.012
	RML	12.356 (9.659)	26.318 (14.621)	0.004	0.016
	RLL	3.316 (5.033)	9.097 (6.254)	<0.001	0.004
	Total	4.923 (5.665)	15.207 (10.043)	<0.001	0.003

*Note*: Values are presented as mean (standard deviation, SD).

Abbreviations: Emph%, percent emphysema‐like symptoms in lung segment; fSAD%, percent functional small airway disease‐like symptoms in lung segment; LUL, left upper lobe, LLL, left lower lobe; RUL, right upper lobe; RML, right middle lobe; RLL, right lower lobe.

**TABLE 4:** QCT‐based structural variables including normalized hydraulic diameter (*D*_h_^*^) and wall thickness (*WT* ^*^) for 11 selected airways.

Variable	Region	Group A	Group B	*p*‐Value	*q*‐Value
*D*_h_*	Trachea	1.035 (0.117)	1.191 (0.160)	0.007	0.021
	RMB	0.833 (0.094)	0.926 (0.079)	0.005	0.017
	Bronint	0.624 (0.066)	0.691 (0.056)	0.004	0.016
	TriRLL	0.423 (0.066)	0.447 (0.055)	0.348	0.432
	LMB	0.662 (0.073)	0.755 (0.108)	0.016	0.041
	TriLLB	0.467 (0.051)	0.497 (0.059)	0.116	0.185
	sRUL	0.296 (0.050)	0.331 (0.069)	0.224	0.310
	sRML	0.264 (0.077)	0.292 (0.064)	0.097	0.163
	sRLL	0.280 (0.041)	0.276 (0.039)	0.934	0.947
	sLUL	0.242 (0.039)	0.256 (0.047)	0.461	0.535
	sLLL	0.316 (0.048)	0.346 (0.076)	0.403	0.483
WT*	Trachea	0.996 (0.120)	1.061 (0.097)	0.097	0.163
	RMB	0.891 (0.188)	1.042 (0.095)	0.038	0.082
	Bronint	0.676 (0.064)	0.712 (0.077)	0.251	0.329
	TriRLL	0.611 (0.041)	0.671 (0.075)	0.048	0.097
	LMB	0.710 (0.115)	0.845 (0.193)	0.072	0.130
	TriLLB	0.640 (0.051)	0.704 (0.060)	0.010	0.029
	sRUL	0.595 (0.033)	0.645 (0.075)	0.112	0.183
	sRML	0.570 (0.069)	0.632 (0.059)	0.004	0.016
	sRLL	0.573 (0.038)	0.606 (0.057)	0.128	0.201
	sLUL	0.535 (0.037)	0.578 (0.054)	0.021	0.049
	sLLL	0.589 (0.041)	0.642 (0.069)	0.034	0.076

*Note*: Values are presented as mean (standard deviation, SD).

Abbreviations: Bronint, bronchus intermedius; LMB, left main bronchus; RMB, right main bronchus; sRUL, subgrouped right upper lobe (includes branches RB1 to RB3); sRML, subgrouped right middle lobe (includes branches RB4 and RB5); sRLL, subgrouped right lower lobe (includes branches RB6 to RB10); sLUL, subgrouped left upper lobe (includes branches LB1 to LB5); sLLL, subgrouped left lower lobe (includes LB6 and LB8 to LB10); TriRLL, trifurcation of the right lower lobe, TriLLB, trifurcation of the left lower bronchus.

Table 5 shows the average diameter (*D*_ave_) of segmental and terminal airways for groups A and B. Values for segmental airways were averaged with 18 branches (i.e., LB1–6, LB9–10, RB1–10) in the CT‐resolved regions (Choi et al., 2013; Choi et al., 2014; Choi et al., 2017; Choi, Hoffman, et al., 1985). The parenchymal diameters of the CT‐unresolved regions were then determined using the stochastic approach described in the Methods section. We found that the crosssectional diameters of segmental and terminal airways did not significantly differ between the two groups (*p* = 0.398 and *p* = 0.398, respectively).

**TABLE 5:** Average diameter (*D*_*ave*_) during breathing for 18 segmental airways and terminal airways.

		Group A	Group B	*p*‐Value	*q*‐Value
*D*_*ave*_ (SD), mm	18 segmental airways	4.529 (0.633)	4.780 (0.701)	0.398	0.483
	Terminal airways	0.543 (0.104)	0.590 (0.120)	0.398	0.483

*Note*: Values are presented as mean (standard deviation, SD).

Table 6 presented the degree of airway constriction and dilation ($D_{\mathrm{ave}}^{*}$). This value of $D_{\mathrm{ave}}^{*}$ in Group B was significantly smaller in RUL (*p* = 0.029), RLL (*p* = 0.006), and total lung (*p* = 0.026) at TLC and in LLL (*p* = 0.038) at FRC than those of Group A.

**TABLE 6:** Estimated degree of airway constriction/dilation ($D_{\mathrm{ave}}^{*}$) determined using CT‐resolved airway data for both groups.

FRC			*p*‐Value	*q*‐Value
Lobe	Group A	Group B		
LUL	0.918 (0.107)	0.969 (0.101)	0.166	0.239
LLL	0.927 (0.110)	0.836 (0.123)	0.038	0.075
RUL	0.864 (0.147)	0.809 (0.143)	0.212	0.293
RML	0.873 (0.139)	0.864 (0.137)	0.585	0.638
RLL	0.876 (0.140)	0.881 (0.158)	0.960	0.960
Total	0.919 (0.118)	0.848 (0.132)	0.188	0.265

TLC			*p*‐Value	q‐Value
Lobe	Group A	Group B		
LUL	1.019 (0.104)	1.031 (0.076)	0.631	0.659
LLL	0.981 (0.140)	0.906 (0.141)	0.104	0.171
RUL	0.995 (0.126)	0.901 (0.135)	0.029	0.063
RML	0.968 (0.119)	0.902 (0.172)	0.120	0.183
RLL	1.042 (0.170)	0.873 (0.167)	0.006	0.018
Total	1.017 (0.135)	0.918 (0.130)	0.026	0.059

*Note*: Values are presented as mean (standard deviation, SD).

Abbreviations: FRC, functional residual capacity, LUL, left upper lobe, LLL, right upper lobe, RUL, right upper lobe, RML, right middle lobe, RLL, right lower lobe; TLC, total lung capacity.

### 3.3 1D CFD analysis

**FIGURE 3:**
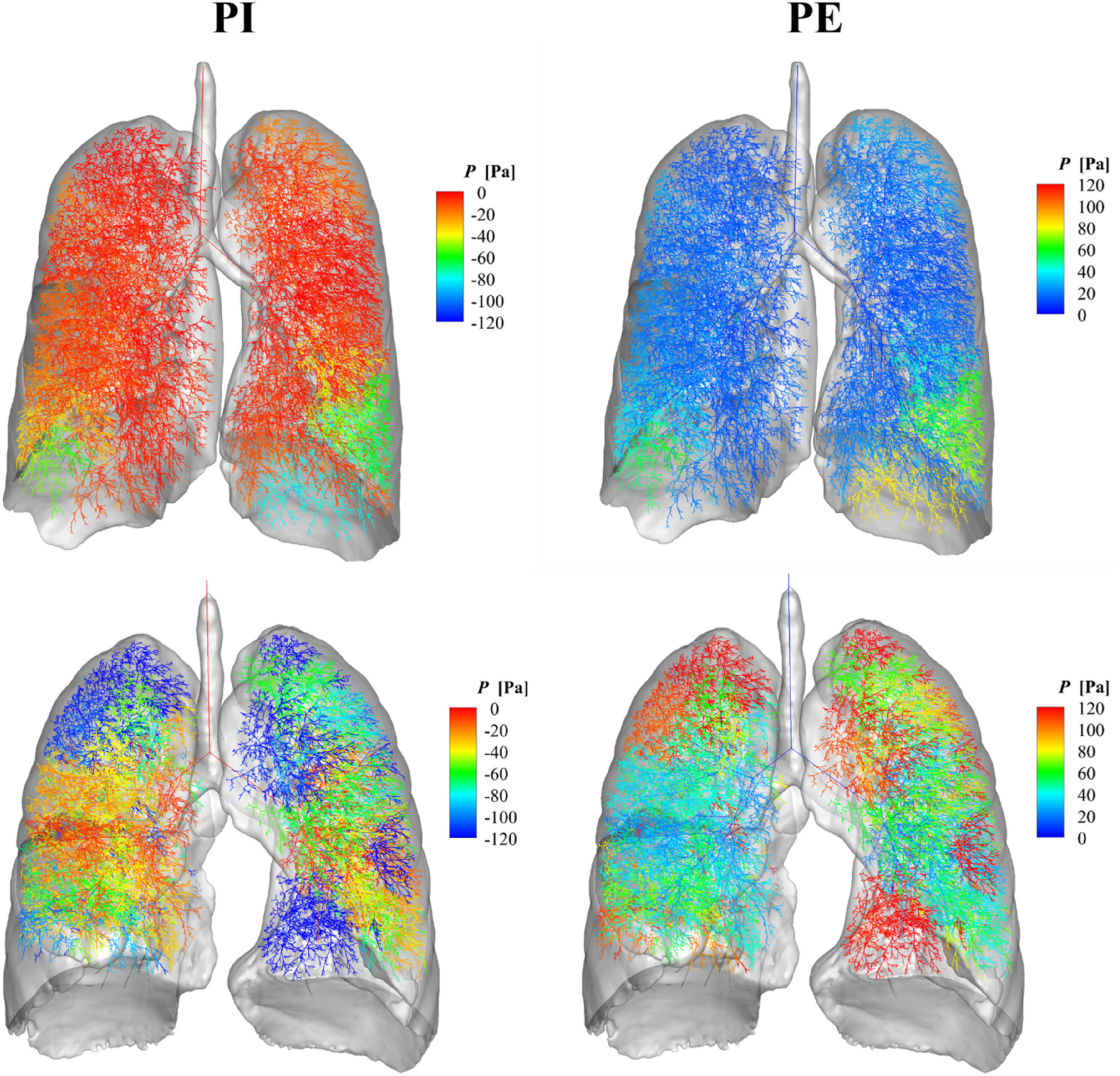
Pressure distributions at peak inspiration (PI) and expiration (PE) of participants with and without FAO. Top: Group A participants (without FAO); bottom: Group B participants (with FAO).

To analyze the regional pressure distribution, we obtained the pleural pressure (*P*_*pl*_), alveolar pressure (*P*_*alv*_), and transpulmonary pressure (*P*_*tp*_) for all participants of each group at critical breathing points, that is, peak inspiration (PI) and peak expiration (PE) (Table 7). *P*_*alv*_ and *P*_*tp*_ were averaged in distal nodes of terminal branches. Note that alveolar pressure is relative to tracheal pressure; hence, it represents a pressure drop along the airway. We found no significant difference in *P*_*alv*_ at both PI (*p* = 0.137) and PE (*p* = 0.608). In contrast, *P*_*pl*_ and *P*_*tp*_ of participants in Group B were greater than those in Group A at both PI (*p* < 0.001 and *p* < 0.001, respectively) and PE (*p* < 0.001 and *p* < 0.001, respectively). To visualize the regional pressure distribution, we selected participants based on the mean postbronchodilator FEV_1_ value (Figure 3) to visualize regional pressure distributions. Overall, the participants from Group B had a higher pressure drop during both inspiration and expiration compared with to ones from Group A. A full animation of breathing can be found at the following links: with FAO (https://youtu.be/W‐RZZg95_IU); without FAO (https://youtu.be/jIU‐pLA3KPU).

**TABLE 7:** Simulated regional pressures (*P*_*alv*_, *P*_*pl*_, *and P*_*tp*_) at peak inspiration and expiration.

Regional pressure	Time	Group A	Group B	*p*‐Value	*q*‐Value
*P*_*alv*_, Pa	PI	−53.254 (37.408)	−74.671 (38.654)	0.137	0.201
	PE	69.560 (41.308)	71.240 (30.653)	0.608	0.653
‐*P*_*pl*_, Pa	PI	924.532 (174.974)	1298.827 (289.789)	<0.001	0.001
	PE	790.229 (180.643)	1295.052 (289.653)	<0.001	<0.001
*P*_*tp*_, Pa	PI	871.277 (173.133)	1137.875 (287.662)	<0.001	0.010
	PE	859.788 (172.016)	1209.115 (285.050)	<0.001	0.003

*Note*: Values are presented as mean (standard deviation, SD).

Abbreviations: *P*_*alv*,_ alveolar pressure; *P*_*pl*,_ pleural pressure; *P*_*tp*,_ transpulmonary pressure, PI, peak inspiration; PE, peak expiration.

### 3.4 Spearman correlation test between PFT variables and 1D CFD outputs

**FIGURE 4:**
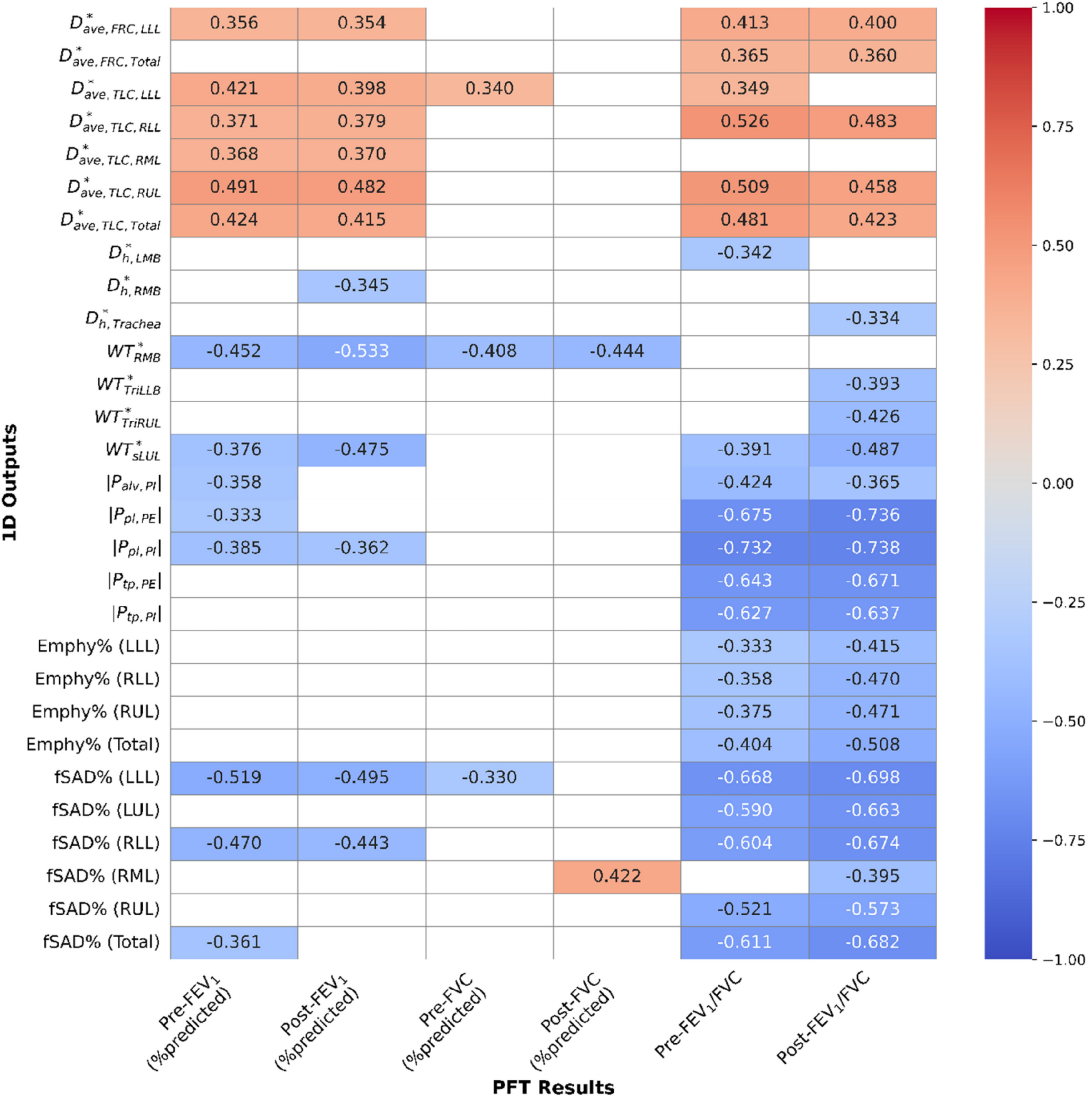
Correlation map between pulmonary function test results and 1D computational outputs and QCT‐based functional variables.

Spearman's correlation analyses were performed for PFT results with metrics from CT‐based imaging and computational simulations. The statistically significant correlations were colored and annotated in Figure 4. Except for segmental and terminal airway diameters, all other computational variables were found to be correlated with pulmonary function measurements. Regarding the computed pressures, the pre‐ and post‐FEV_1_ were negatively correlated with *P*_*pl*_ at PI (*ρ* = −0.385 and *ρ* = −0.362, respectively). The *P*_*pl*_ at PE and *P*_*alv*_ at PI were negatively correlated with only the pre‐FEV_1_ (*ρ* = −0.333 and *ρ* = −0.358, respectively). The pre‐ and post‐FVC values showed no correlations with 1D CFD outputs. The FEV_1_/FVC ratio was a crucial classification factor for FAO. Note that the pre‐ and post‐ values of FEV_1_/FVC were observed to be negatively correlated with *P*_*pl*_ at PI (*ρ* = −0.732, and *ρ* = −0.738, respectively) and PE (*ρ* = −0.675, and *ρ* = −0.736, respectively), and *P*_*tp*_ at PI (*ρ* = −0.627, and ρ = −0.637, respectively) and PE (*ρ* = −0.643, and *ρ* = −0.671, respectively), and *P*_*alv*_ at PI (*ρ* = −0.424, and *ρ* = −0.365, respectively).

The Emph% and fSAD% also showed correlations with the FEV_1_/FVC ratio. The pre‐ and post‐FEV_1_/FVC was negatively correlated with Emph% in LLL (*ρ* = −0.333, and *ρ* = −0.415, respectively), RLL (*ρ* = −0.358, and *ρ* = −0.470, respectively), RUL (*ρ* = −0.375, and *ρ* = −0.471, respectively), and total lung (*ρ* = −0.404, and *ρ* = −0.508, respectively). The fSAD% were negatively correlated with both pre‐ and post‐FEV_1_/FVC with ρ ≤ −0.6 in all lung regions except RML.

Positive correlations were observed between the degree of constriction $D_{\mathrm{ave}}^{*}$ with FEV_1_ and FEV_1_/FVC ratio. While $D_{\mathrm{ave}}^{*}$ only in LLL was correlated with both pre‐ and post‐FEV_1_ at FRC (*ρ* = −0.356, and ρ = −0.354, respectively), it showed better correlations in all lung regions at TLC. The pre‐ and post‐FEV_1_/FVC ratios were correlated with $D_{\mathrm{ave}}^{*}$ in some regions including RLL (*ρ* = −0.526, and *ρ* = −0.483, respectively), RUL (*ρ* = −0.509, and *ρ* = −0.458, respectively), total lung (*ρ* = −0.481, and *ρ* = −0.423, respectively) at TLC and LLL (*ρ* = −0.413, and *ρ* = −0.400, respectively), total lung (*ρ* = −0.365, and *ρ* = −0.360, respectively) at FRC.

## 4 DISCUSSION

In the current study, we investigated the airway structural and functional characteristics of two asthmatic groups, one with FAO and the other with reversible airway obstruction. This study included Koreans with asthma in a clinical setting and classified them according to their lung function recovery. Several studies have investigated the differences between healthy and asthma groups and between groups with severe and non severe asthma (Choi et al., 2013; Choi et al., 2018; Choi, Hoffman, et al., 1985; Jahani et al., 2017; Kim et al., 2015). However, to the best of our knowledge, this is the first effort to apply an integrated set of imaging variables with 1D computational methods to the FAO verses non‐FAO participants. We extracted several QCT structural variables and used image registration to derive Emph% and fSAD% values for all participants. More importantly, we used a 1D CFD model to investigate airway pressure distributions. Furthermore, we derived a new measure of healthy $D_{\mathrm{ave}}^{r}$, as per lung lobes and generations for Korean participants. This metric could assess subject‐specific airway constriction and dilation. We used the metric for generating diameters of CT‐unresolved airways for the 1D CFD simulations.

Since Group B is characterized by chronic alteration in PFT, we found that overall PFT metrics were clearly lower in Group B than Group A, as expected. In terms of CT‐based structural variables, after normalizing with sex‐height‐age‐based predicted trachea diameters, normalized hydraulic diameters in trachea, RMB, Bronint, and LMB regions were significantly larger in the participants in Group B. Note that those regions do not belong to the lung lobes. We also found that the wall thickness was significantly larger in RMB, TriRLL, TriLLB, sRML, sLUL, and sLLL regions in participants in Group B. Several studies demonstrated that participants with SA are characterized by increased wall area percent, decreased lumen area, more airway narrowing, and more pruning of the peripheral pulmonary vasculature (Choi, Hoffman, et al., 1985). We believe that these features could be similarly found in a larger number of samples than the current study.

Previous studies (Bell et al., 2019; Park et al., 2021) demonstrated that an increase of fSAD% is significantly correlated with ventilation heterogeneity. Additionally, the involvement of small airways in asthma can worsen disease symptoms in participants with asthma, so that therapies targeting these narrow regions can result in lung function improvement (Carr et al., 2017; Cohen et al., 2008). Interestingly, we also found fSAD% was significantly greater in participants with FAO, especially in the lower lobes. Emphysema could be causative for many symptoms such as hyperinflation, airflow obstruction, gas exchange abnormalities. A series of computed tomographic scans demonstrated that the occurrence of small airway obstruction often develops emphysema afterwards (Galbán et al., 2012). However, even with mild airflow obstruction, emphysema could exist in participants with COPD (Postma et al., 2015). A more extensive focus on emphysema is needed to describe their associations with asthma with FAO.

Airway diameter, pressure distribution, and hysteresis curves in the segmental and peripheral airway regions were assessed via 1D lung CFD simulations considering the dynamic deformation of lung diameter and length. The intrapleural and transpulmonary pressures were much greater in Group B at both PI and PE. The intrapleural pressures were determined by alveolar pressure, alveolar volume, and regional compliance, where the alveolar pressure was estimated by pressure drop from trachea to terminal bronchioles (Wongviriyawong et al., 2013). In the current study, we set the same global lung compliance among the participants, so a decrease of pleural pressure results from the larger lung volume in Group B which is a consequence of residual gas and air‐trapping at FRC (increased fSAD%). The smaller *P*_*alv*_ also yields a significant decrease of *P*_*pl*_. We conjecture that the larger pressure drops and residual gas in the terminal airways in participants with FAO could result in higher pleural pressures. A larger cohort of FAO is needed to analyze further to answer this question in the future. Previously, Choi et al. (Choi, Yoon, et al., 1985) estimated the ratio of average diameter and respective predicted diameter ($D_{\mathrm{ave}}^{*}$) as airway constriction, and they found that participants with asthma showed more constricted airways than healthy participants in US populations. In this study, we newly measured diameters of CT‐resolved airways in 222 healthy Korean participants, and derived healthy $D_{\mathrm{ave}}^{r}$ for assessing airway constriction or dilation. The $D_{\mathrm{ave}}^{r}$ was used to obtain subject‐specific $D_{\mathrm{ave}}^{*}$ as per lung lobes. This approach imposed random heterogeneity based on lobar mean and standard deviation of $D_{\mathrm{ave}}^{*}$ to apply nonuniform constriction for the CT‐unresolved airway diameters. Consequently, we found that Group B had higher levels of airway constriction than Group A, which could cause higher pressure drops in pleural regions in Group B.

**FIGURE 5:**
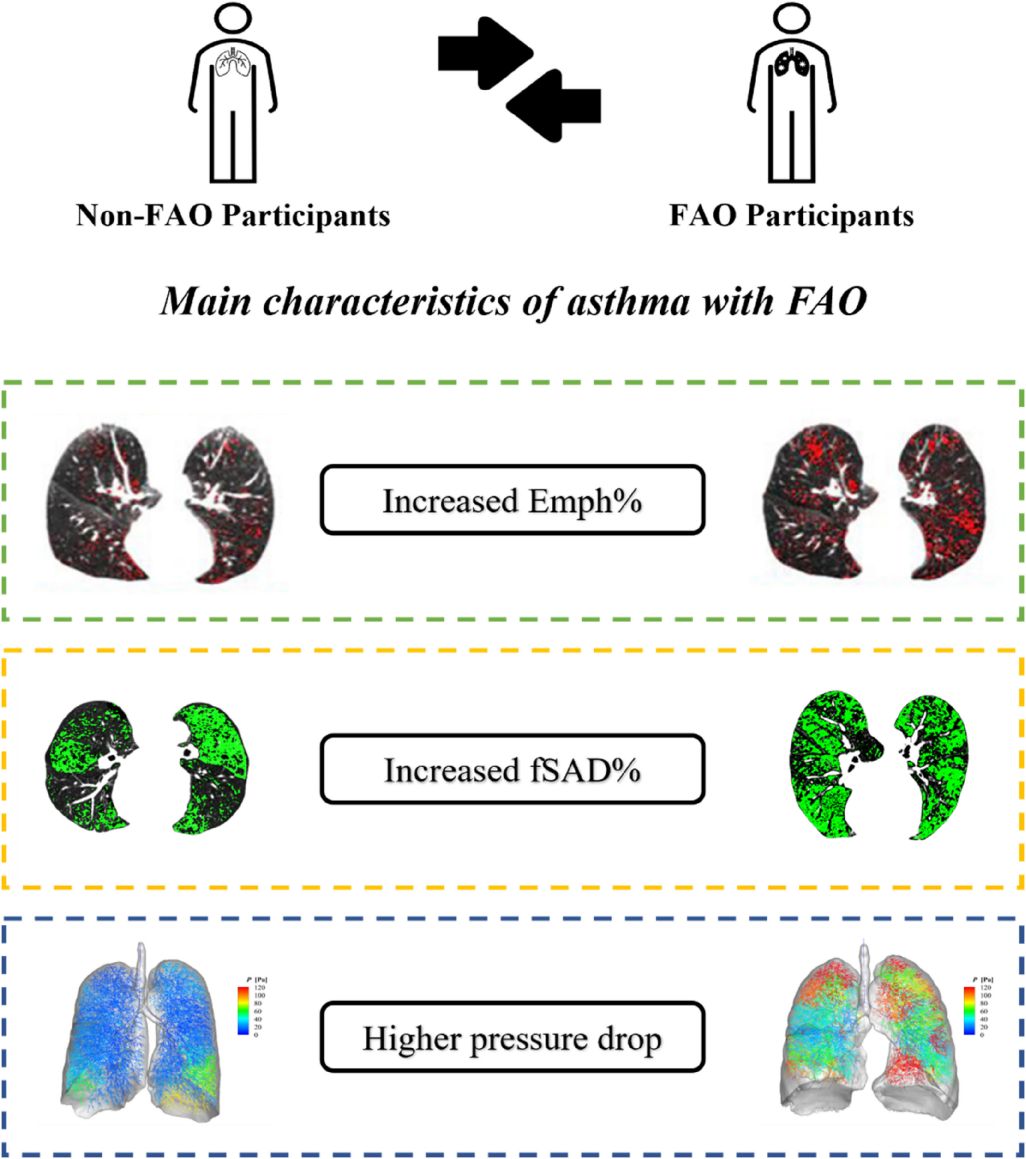
Major alterations associated with FAO as determined using imaging‐based and computational modeling.

The computational parameters were strongly correlated with pulmonary function measures. We observed that the intrapleural and intrapulmonary pressure during inspiration had a considerable correlation with the PFT results, specifically with FEV_1_. In particular, intrapleural pressures increase with decreasing FEV_1_ (%predicted) only during inspiration while Group B had significantly higher absolute magnitudes of this parameter compared to those in Group A. This indicates that FAO populations could experience malfunctions in lung inflation. The FEV_1_/FVC ratio, which is used to classify the two groups, has significantly negative correlations with all the computational pressures at PI and PE, except for alveolar pressure at PE. These correlations prove the considerable relations between the pressure drops and the FAO disease, however, this finding should be validated with a larger cohort in the future. In the meantime, the major alterations in FAO participants are summarized in Figure 5.

This study has certain limitations. First, this study is retrospectively designed, so the sample size of participants with FAO was smaller than that of the reversible group; this can potentially affect statistical reliability. Additionally, the number of samples was quite small and there were significantly more female participants in Group A than Group B. We suggest an analysis of a larger cohort to further investigate the sex effect in the future. Moreover, we imposed the same inlet flow rate as well as same compliance for all participants owing to the lack of subject‐specific breathing pattern and tissue properties; however, this information may provide the fair comparison of participants with fixed and reversible airway obstructive asthma by only considering the effect of anatomical data of airways. Additionally, CT‐unresolved diameters were stochastically estimated. Unless higher resolution is applicable, this method could be considered a good approximation. With all the limitations considered, the presented 1D CFD data may be useful for identifying next‐generation biomarkers of asthma with FAO for a sufficiently large number of samples in the future.

In conclusion, the fixed airway obstructive population included in this study presented differences in lung function and pressure distribution in small airways as determined through PFTs, QCT‐based metrics, and 1D CFD simulation. Participants with FAO have greater pressure drops in the terminal regions which could be the result of higher degree of airway constrictions. Using the 1D CFD model to compare the two groups, we identified significant differences in terms of pressure distribution and strong correlations between 1D and PFT results. Taken together, this approach may contribute to a better understanding of the mechanisms underlying FAO and support the treatment of this type of disease in the future.

## AUTHOR CONTRIBUTIONS

Q.H.N., S.C., G.Y.J: Conceptualization, Methodology, Software, Validation. Q.H.N., S.C., G.Y.J., S.R.K., K.J.C: Resources, Data curation, Writing‐ Original draft preparation, Writing‐ Review & Editing. S.C., G.Y.J: Supervision.

## FUNDING INFORMATION

This study was supported by the DongKook Life Science Co., Ltd, Republic of Korea.

## Ethics Statement

This study was retrospectively designed and approved by the Institutional Review Board of Jeonbuk National University Hospital (IRB‐2019‐07‐032‐008). The participant consent forms were obtained verbally when participant imaging and clinical data are obtained. Since this study was designed retrospectively, the written informed consent form was waived by the IRB.

## Supporting information


Table S1.
