# Supplementary material for: Structural and functional features of asthma participants with fixed airway obstruction using CT imaging and 1D computational fluid dynamics: A feasibility study
Source: Physiol Rep. 2024 Jan 7;12(1):e15909. doi: 10.14814/phy2.15909 (PMC10771932; doi:10.14814/phy2.15909)
Supplement: Supplementary file 1 — Table S1. [file PHY2-12-e15909-s001.docx]

**Supplemental Table S1.** *Correlation table between pulmonary function test results and 1D computational outputs and QCT-based functional variables.*

|  | Pre-FEV_1_ (%predicted) | Post-FEV_1_ (%predicted) | Pre-FVC (%predicted) | Post-FVC (%predicted) | Pre-FEV_1_/FVC (%predicted) | Post-FEV_1_/FVC (%predicted) |
| --- | --- | --- | --- | --- | --- | --- |
| D^*^_ave, FRC, LLL_ | 0.356 (0.033) | 0.354 (0.037) | 0.232 (0.174) | 0.239 (0.166) | 0.413 (0.012) | 0.400 (0.017) |
| D^*^_ave, FRC, LUL_ | 0.063 (0.715) | -0.011 (0.948) | 0.035 (0.842) | 0.033 (0.853) | -0.055 (0.752) | -0.110 (0.528) |
| D^*^_ave, FRC, RLL_ | 0.154 (0.370) | 0.138 (0.429) | 0.021 (0.903) | 0.088 (0.617) | 0.193 (0.260) | 0.109 (0.533) |
| D^*^_ave, FRC, RML_ | 0.035 (0.841) | -0.003 (0.986) | -0.046 (0.790) | 0.005 (0.979) | 0.156 (0.363) | 0.123 (0.482) |
| D^*^_ave, FRC, RUL_ | 0.171 (0.318) | 0.194 (0.265) | 0.118 (0.493) | 0.159 (0.362) | 0.245 (0.150) | 0.252 (0.144) |
| D^*^_ave, FRC, Total_ | 0.254 (0.135) | 0.170 (0.328) | 0.175 (0.307) | 0.091 (0.603) | 0.365 (0.029) | 0.360 (0.034) |
| D^*^_ave, TLC, LLL_ | 0.421 (0.011) | 0.398 (0.018) | 0.340 (0.042) | 0.306 (0.074) | 0.349 (0.037) | 0.309 (0.071) |
| D^*^_ave, TLC, LUL_ | 0.080 (0.641) | -0.002 (0.991) | 0.014 (0.938) | -0.119 (0.495) | 0.030 (0.862) | 0.004 (0.981) |
| D^*^_ave, TLC, RLL_ | 0.371 (0.026) | 0.379 (0.025) | 0.104 (0.547) | 0.106 (0.545) | 0.526 (0.001) | 0.483 (0.003) |
| D^*^_ave, TLC, RML_ | 0.368 (0.027) | 0.370 (0.029) | 0.214 (0.209) | 0.249 (0.149) | 0.324 (0.054) | 0.268 (0.119) |
| D^*^_ave, TLC, RUL_ | 0.491 (0.002) | 0.482 (0.003) | 0.322 (0.055) | 0.318 (0.063) | 0.509 (0.002) | 0.458 (0.006) |
| D^*^_ave, TLC, Total_ | 0.424 (0.010) | 0.415 (0.013) | 0.226 (0.184) | 0.215 (0.215) | 0.481 (0.003) | 0.423 (0.011) |
| D^*^_h, Bronint_ | -0.236 (0.166) | -0.308 (0.072) | -0.258 (0.128) | -0.284 (0.098) | -0.302 (0.073) | -0.296 (0.084) |
| D^*^_h, LMB_ | -0.267 (0.115) | -0.324 (0.058) | -0.271 (0.110) | -0.291 (0.090) | -0.342 (0.041) | -0.321 (0.060) |
| D^*^_h, RMB_ | -0.279 (0.099) | -0.345 (0.042) | -0.273 (0.107) | -0.263 (0.127) | -0.306 (0.070) | -0.325 (0.057) |
| D^*^_h, Trachea_ | -0.284 (0.094) | -0.303 (0.077) | -0.292 (0.084) | -0.230 (0.184) | -0.316 (0.060) | -0.334 (0.050) |
| D^*^_h, TriLLB_ | -0.096 (0.601) | -0.059 (0.755) | -0.043 (0.816) | 0.073 (0.695) | -0.197 (0.279) | -0.213 (0.250) |
| D^*^_h, TriRLL_ | 0.249 (0.176) | 0.214 (0.256) | 0.222 (0.231) | 0.213 (0.259) | 0.202 (0.275) | 0.102 (0.591) |
| D^*^_h, TriRUL_ | 0.106 (0.558) | 0.005 (0.980) | 0.138 (0.443) | 0.150 (0.412) | -0.191 (0.287) | -0.285 (0.114) |
| D^*^_h, sLLL_ | 0.212 (0.230) | 0.201 (0.262) | 0.223 (0.206) | 0.269 (0.130) | 0.076 (0.671) | 0.002 (0.991) |
| D^*^_h, sLUL_ | -0.030 (0.863) | -0.095 (0.593) | -0.117 (0.503) | -0.130 (0.464) | -0.010 (0.955) | -0.068 (0.703) |
| D^*^_h, sRLL_ | 0.266 (0.117) | 0.249 (0.149) | 0.149 (0.385) | 0.195 (0.262) | 0.245 (0.149) | 0.172 (0.324) |
| D^*^_h, sRML_ | 0.170 (0.322) | 0.141 (0.420) | 0.120 (0.487) | 0.195 (0.261) | 0.021 (0.906) | -0.112 (0.522) |
| D^*^_h, sRUL_ | 0.037 (0.828) | 0.062 (0.725) | 0.081 (0.640) | 0.215 (0.215) | 0.008 (0.964) | -0.015 (0.932) |
| D_segmental_ | 0.082 (0.635) | 0.021 (0.903) | 0.022 (0.900) | 0.069 (0.695) | 0.091 (0.598) | -0.016 (0.927) |
| D_terminal_ | -0.072 (0.677) | -0.107 (0.539) | -0.175 (0.307) | -0.103 (0.556) | 0.021 (0.902) | -0.032 (0.857) |
| WT^*^_Bronint_ | -0.153 (0.372) | -0.200 (0.249) | -0.174 (0.309) | -0.222 (0.201) | -0.102 (0.553) | -0.091 (0.602) |
| WT^*^_LMB_ | -0.309 (0.067) | -0.324 (0.058) | -0.306 (0.069) | -0.267 (0.121) | -0.321 (0.056) | -0.309 (0.071) |
| WT^*^_RMB_ | -0.452 (0.006) | -0.533 (0.001) | -0.408 (0.014) | -0.444 (0.008) | -0.321 (0.057) | -0.331 (0.052) |
| WT^*^_Trachea_ | -0.217 (0.203) | -0.217 (0.210) | -0.125 (0.469) | -0.156 (0.370) | -0.273 (0.107) | -0.239 (0.167) |
| WT^*^_TriLLB_ | -0.188 (0.304) | -0.206 (0.267) | -0.080 (0.665) | -0.122 (0.512) | -0.337 (0.059) | -0.393 (0.029) |
| WT^*^_TriRLL_ | -0.183 (0.324) | -0.238 (0.205) | -0.142 (0.446) | -0.218 (0.246) | -0.267 (0.146) | -0.288 (0.123) |
| WT^*^_TriRUL_ | -0.194 (0.279) | -0.295 (0.101) | -0.062 (0.730) | -0.166 (0.363) | -0.324 (0.066) | -0.426 (0.015) |
| WT^*^_sLLL_ | -0.115 (0.519) | -0.170 (0.345) | -0.019 (0.914) | -0.070 (0.700) | -0.224 (0.202) | -0.288 (0.104) |
| WT^*^_sLUL_ | -0.376 (0.026) | -0.475 (0.005) | -0.277 (0.107) | -0.338 (0.051) | -0.391 (0.020) | -0.487 (0.004) |
| WT^*^_sRLL_ | -0.154 (0.371) | -0.215 (0.215) | -0.043 (0.802) | -0.095 (0.588) | -0.071 (0.682) | -0.105 (0.547) |
| WT^*^_sRML_ | -0.202 (0.237) | -0.214 (0.216) | -0.141 (0.412) | -0.105 (0.548) | -0.207 (0.225) | -0.251 (0.145) |
| WT^*^_sRUL_ | -0.258 (0.128) | -0.317 (0.063) | -0.180 (0.292) | -0.206 (0.234) | -0.207 (0.226) | -0.224 (0.197) |
| $\left\vert\text{P}_{\text{alv}\text{, P}\text{E}} \right\vert$ | -0.216 (0.205) | -0.168 (0.335) | -0.057 (0.743) | -0.049 (0.779) | -0.247 (0.146) | -0.187 (0.281) |
| $\left\vert\text{P}_{\text{alv}\text{, PI}} \right\vert$ | -0.358 (0.032) | -0.332 (0.052) | -0.158 (0.357) | -0.172 (0.324) | -0.424 (0.010) | -0.365 (0.031) |
| $\left\vert\text{P}_{\text{pl}\text{, P}\text{E}} \right\vert$ | -0.333 (0.047) | -0.326 (0.056) | -0.118 (0.493) | 0.014 (0.936) | -0.675 (0.000) | -0.736 (0.000) |
| $\left\vert\text{P}_{\text{pl}\text{, PI}} \right\vert$ | -0.385 (0.020) | -0.362 (0.032) | -0.180 (0.294) | -0.054 (0.760) | -0.732 (0.000) | -0.738 (0.000) |
| $\left\vert\text{P}_{\text{tp}\text{, P}\text{E}} \right\vert$ | -0.312 (0.064) | -0.272 (0.114) | -0.133 (0.441) | 0.039 (0.823) | -0.643 (0.000) | -0.671 (0.000) |
| $\left\vert\text{P}_{\text{tp}\text{, PI}} \right\vert$ | -0.301 (0.074) | -0.266 (0.123) | -0.131 (0.445) | 0.017 (0.922) | -0.627 (0.000) | -0.637 (0.000) |
| Emphy% (LLL) | -0.215 (0.207) | -0.266 (0.122) | -0.115 (0.503) | -0.063 (0.718) | -0.333 (0.048) | -0.415 (0.013) |
| Emphy% (LUL) | -0.040 (0.815) | -0.059 (0.737) | -0.092 (0.592) | -0.174 (0.318) | 0.004 (0.980) | 0.008 (0.962) |
| Emphy% (RLL) | -0.109 (0.528) | -0.122 (0.483) | 0.002 (0.990) | 0.099 (0.570) | -0.358 (0.032) | -0.470 (0.004) |
| Emphy% (RML) | -0.017 (0.920) | -0.026 (0.884) | 0.018 (0.916) | 0.088 (0.613) | -0.129 (0.454) | -0.229 (0.187) |
| Emphy% (RUL) | -0.088 (0.608) | -0.080 (0.649) | 0.018 (0.917) | 0.115 (0.509) | -0.375 (0.024) | -0.471 (0.004) |
| Emphy% (Total) | -0.191 (0.264) | -0.210 (0.225) | -0.069 (0.690) | 0.034 (0.848) | -0.404 (0.015) | -0.508 (0.002) |
| fSAD% (LLL) | -0.519 (0.001) | -0.495 (0.003) | -0.330 (0.049) | -0.221 (0.202) | -0.668 (0.000) | -0.698 (0.000) |
| fSAD% (LUL) | -0.323 (0.055) | -0.229 (0.186) | -0.180 (0.295) | 0.016 (0.928) | -0.590 (0.000) | -0.663 (0.000) |
| fSAD% (RLL) | -0.470 (0.004) | -0.443 (0.008) | -0.265 (0.118) | -0.143 (0.414) | -0.604 (0.000) | -0.674 (0.000) |
| fSAD% (RML) | -0.014 (0.933) | 0.081 (0.645) | 0.222 (0.194) | 0.422 (0.011) | -0.291 (0.085) | -0.395 (0.019) |
| fSAD% (RUL) | -0.251 (0.140) | -0.177 (0.310) | -0.077 (0.655) | 0.092 (0.600) | -0.521 (0.001) | -0.573 (0.000) |
| fSAD% (Total) | -0.361 (0.030) | -0.308 (0.072) | -0.140 (0.416) | 0.023 (0.894) | -0.611 (0.000) | -0.682 (0.000) |

Values are presented as Pearson correlation coefficient (p value).
